# Recent Developments in Vaccines against Flaviviruses and Alphaviruses

**DOI:** 10.3390/vaccines11020448

**Published:** 2023-02-15

**Authors:** Young Chan Kim, Arturo Reyes-Sandoval

**Affiliations:** 1 The Jenner Institute, Nuffield Department of Medicine, University of Oxford, Roosevelt Drive, Oxford OX3 7DG, UK; 2 Instituto Politécnico Nacional (IPN), Av. Luis Enrique Erro s/n., Unidad Adolfo López Mateos, México City 07738, Mexico

In the twenty-first century, newly emerging viruses which are mostly zoonotic or vector-borne have continuously threatened public health and caused outbreaks of global concern. This has been highlighted by the recent COVID-19 pandemic which caused a catastrophic effect on the world’s healthcare system and the global economy [1]. Flaviviruses and alphaviruses are single-stranded RNA viruses vectored by *Aedes* mosquitoes that can (re)-emerge unexpectedly and cause severe viral infections in humans [2,3,4,5,6,7,8]. These flaviviruses and alphaviruses can be classified into a broader category of arboviruses, and they cause significant disease burdens and public health concerns due to the global spread and transmission over the last century [6,7,8,9,10].

The mosquito-borne flaviviruses such as dengue virus (DENV), Zika virus (ZIKV), yellow fever virus (YFV), West Nile virus (WNV), and Japanese encephalitis virus (JEV) are responsible for significant human morbidity and mortality all over the world [7,8,11,12]. In particular, DENV is estimated to cause around 400 million infections annually and 20% of infections lead to 22,000 deaths per year with more than a quarter of the world’s population now living in DENV-endemic areas [13,14,15]. The rapid geographical introduction and spread of WNV and ZIKV from the Eastern to the Western Hemisphere had caused a large number of cases with significant morbidity [16,17]. Although most ZIKV infections are asymptomatic, some ZIKV infections are associated with congenital Zika syndrome (CZS) and Guillain–Barré Syndrome (GBS) [18,19,20]. Despite the existence of a highly effective YFV vaccine, the re-emergence of YFV throughout Africa and the Americas now poses a serious public health challenge [21,22].

The alphaviruses are a genus of enveloped RNA viruses with medically important alphaviruses such as Chikungunya virus (CHIKV), Mayaro virus (MAYV), and Eastern equine encephalitis virus (EEEV) that can cause arthralgia (CHIKV and MAYV) or neuroinvasive disease (EEEV) [5,9,10,23]. Following the first identification of CHIKV in Tanzania in 1952 and subsequently, in Africa and Asia, CHIKV outbreaks became more prevalent since 2004, and CHIKV cases are now reported in over 100 countries in Asia, Africa, Europe, and the Americas [10,24,25]. For alphavirus infections, there are no specific antiviral drugs or licensed vaccines, and therefore, the current treatment is mainly symptom relief.

Despite continued threats from emerging viral diseases, the COVID-19 pandemic has demonstrated that the twenty-first century has come with a new era in vaccinology in which recombinant genetic technologies allowed remarkably rapid development of vaccines against SARS-CoV-2 in 2020 [26]. Over the last century, many vaccines based on classical platforms have played a major role in eradicating diseases such as polio and smallpox (Figure 1) [27,28]. However, the speed of the vaccine developments using the classical platforms is considerably slower than the next-generation platforms as these platforms are not often optimized for rapid large-scale production due to unavoidable limitations such as the requirement of biosafety level 3 conditions to grow large quantities of viruses for production of virus-inactivated vaccine and the extensive safety assessment and contraindications associated with administration of live-attenuated vaccines to immunocompromised and pregnant individuals [29,30]. On the other hand, the development of next-generation vaccines can go ahead as soon as the viral sequence becomes known and the DNA sequence of the whole or part of viral antigens with the critical epitopes can be used to develop vaccines using recombinant DNA technologies and thus significantly speeding up vaccine development [31,32]. These next-generation vaccine platforms include viral vectors, nucleic acid (DNA or RNA), and antigen-presenting cells (APC) (Figure 1) [26,30,33]. The potential of next-generation vaccine platforms was clearly shown during the COVID-19 pandemic where the fastest vaccine candidates that reached the phase I clinical trials were based on the next-generation platforms such as mRNA, DNA, human adenoviral vector, and chimpanzee adenoviral vector (ChAdOx1) [26]. In particular, the mRNA vaccine candidate had set a record time by reaching the clinical trial in only 69 days after the identification of the SARS-CoV-2 [26,34]. This pace of vaccine development was striking when compared to arboviral diseases caused by flaviviruses and alphaviruses such as DENV [35], CHIKV [25,36], and ZIKV [37,38,39] which reached trials in 52, 19, and 9 years after the declaration of major outbreaks by WHO, respectively as we discussed previously [26,37,40]. Looking on the bright side, the time taken for these vaccines to reach trials has progressively become shortened with the development of recombinant genetic technology. For instance, the first Zika vaccine candidate to reach the clinical trial in August 2016 was a DNA vaccine candidate which was 9 years after the ZIKV outbreak in Micronesia [37], 3 years after the major epidemic in French Polynesia in 2013 [41] but just 6 months after WHO declared Zika-related microcephaly as a Public Health Emergency of International Concern (PHEIC) in February 2016 [42] highlighting the advances in the modern vaccine development in urgent need [26].

This Special Issue will feature the recent development of vaccines against flaviviruses and alphaviruses. Although licensed vaccines against flaviviruses exist such as DENV (tetravalent vaccine), JEV (inactivated), tick-borne encephalitis (inactivated), and YFV (attenuated), there are currently no vaccines against WNV and ZIKV [43]. Moreover, there may be a need for next-generation vaccines to overcome potential problems with an insufficient supply of inactivated vaccines and contraindications associated with the administration of live-attenuated vaccines to immunocompromised and pregnant individuals [22,43]. Despite slow progress in vaccine development against alphaviruses, there are currently many promising vaccines in clinical trials based on both the classical and next-generation platforms which could lead to the future licensing of vaccines against these medically important emerging arboviruses.

## Figures and Tables

**Figure 1:**
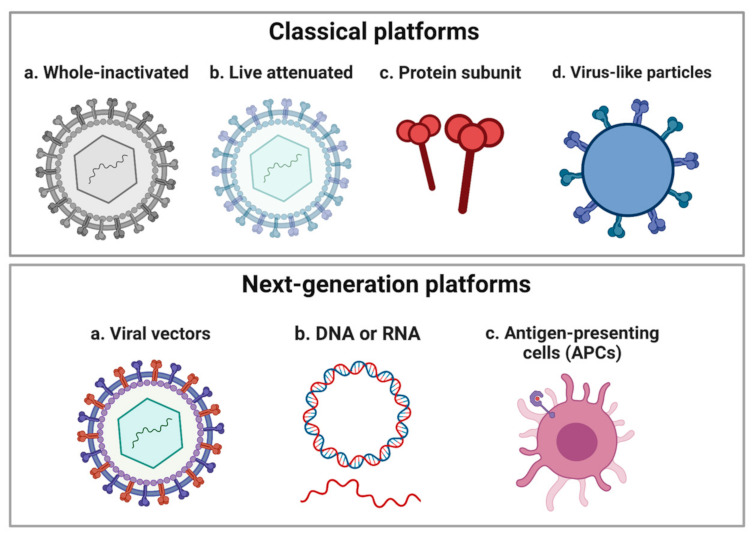
A schematic showing the classical vaccine platforms and next-generation platforms. The classical platforms include whole-inactivated, live attenuated, protein subunit, and virus-like particles (VLPs). The next-generation platforms include recombinant viral vectors, nucleic acid (DNA or RNA), and antigen-presenting cells (APC). Created with BioRender.com.
